# The factor structure of the Motivated Strategies for Learning Questionnaire (MSLQ): new methodological approaches and evidence

**DOI:** 10.1186/s41155-023-00280-0

**Published:** 2023-12-07

**Authors:** Jhonys de Araujo, Cristiano Mauro Assis Gomes, Enio Galinkin Jelihovschi

**Affiliations:** 1 Department of Psychology, Laboratory for Cognitive Architecture Mapping (LAICO), Universidade Federal de Minas Gerais (UFMG), Campus Pampulha. Av. Pres. Antônio Carlos, 6627 – Pampulha, Belo Horizonte, MG CEP: 31270-901 Brazil; 2 Department of Exact and Technological Sciences, Universidade Estadual de Santa Cruz (UESC), Campus Soane Nazaré de Andrade. Nazaré de Andrade Rodovia Ilhéus-Itabuna km, Ilhéus, BA CEP: 16.45.662-900 Brazil

**Keywords:** Factor analysis, Questionnaire, Self-regulated learning, Structural validity

## Abstract

**Background:**

The area of self-regulated learning integrates the fields of metacognition and self-regulation and assumes that the student is an active processor of information capable of self-regulating his learning by putting together the cognitive, metacognitive, and motivational components. The Motivated Strategies for Learning Questionnaire (MSLQ) is a benchmark for the measurement of self-regulated learning. However, the field of study does not show adequate evidence of its structural validity. The vast majority of studies involving this question present serious methodological mistakes, compromising the evidence of validity.

**Objective:**

Our study investigates the structural validity of MSLQ including all 15 scales and corrects relevant mistakes in the previous studies.

**Method:**

We tested different models through item confirmatory factor analysis in a convenience sample of 670 college students (*M* = 22.8 years, *SD* = 5.2) from a public Brazilian university in the technological area. The models with the ML, MLR, MLM and WLMSV estimators.

**Results:**

Only WLSMV produced models with acceptable fit. The final model has a bi-factor structure with a general factor (self-regulated learning), 15 components as first-order factors, and four broad components as second-order factors. Twelve first-order components, all second-order components and the general factor had acceptable reliability. The components’ elaboration, intrinsic goal orientation and metacognitive self-regulation, did not show acceptable reliability, in terms of McDonald’s omega.

**Conclusion:**

Considering the worldwide importance of the MSLQ, we do not recommend the use of the measurement of these components for clinical practice and psychoeducational diagnosis until new studies show that this low reliability only occurs in our sample. Our study shows new evidence, correcting many previous methodological mistakes and producing initial evidence favorable to the factor structure of the MSLQ.

The construct self-regulated learning emerged in the 1980s when researchers interested in integrating knowledge from the fields of metacognition and self-regulation studies decided to deepen their understanding of academic learning (Schunk & Greene, 2017). Mannion (2018, Impact Articles section) proposes that “self-regulated learning is the application of metacognition and self-regulation to learning”, on the basis that “metacognition is monitoring and controlling your thought processes” and “self-regulation is monitoring and controlling your emotions and behaviors”. Self-regulated learning is defined by the area as the dynamic interaction person-environment-task, necessarily involving three broad components: cognition, metacognition and motivation. The area stipulates that through these components the subject interacts with the objects of knowledge and self-regulates, considering the context of the environment and the demands of the task (Dinsmore et al., 2008).

The Motivated Strategies for Learning Questionnaire (MSLQ) is a traditional, consolidated, and widely used measure of self-regulated learning and has been translated into several languages and applied in various cultural contexts (Duncan & McKeachie, 2005). The reviews by Broadbent and Poon (2015) and Roth et al., (2016), show that the MSLQ was the most widely used self-regulated learning assessment instrument in higher education. The first review shows that the MSLQ was employed in 75% of the 12 studies that assessed self-regulated learning in the context of virtual learning, while the second shows that the MSLQ was used in 62% of the 152 studies reviewed.

The MSLQ is theoretically underpinned by sociocognitive perspective. According to this view, the students are responsible for their own learning process. This conception assumes that the students regulate their environment, emotions, motivations, and strategies to optimize the way they learn (Duncan & McKeachie, 2005; Pintrich et al., 1991).

The MSLQ follows the basic guidelines of the field of studies in self-regulated learning and focuses its measurement on motivational, cognitive, and metacognitive components. The MSLQ has 6 motivation scales and 9 scales of cognitive and metacognitive strategies (see Table 1). These scales are conceptually based on previous theories, such as Bandura’s self-efficacy, Eccles and Wigfield’s expectancy-value, and Deci and Ryan’s self-determination (Nanol, 2015).

**Table 1:** Description of the 15 MSLQ components

Component	Description
Intrinsic goal orientation	Represents how much the student engages in a task because of interest in learning, curiosity, and the pursuit of self-improvement.
Extrinsic goal orientation	It defines how much the student engages in a task for reasons extrinsic to the activity itself, such as rewards, grades, performance, and competition.
Task value	Represents the value that the student attaches to a task, defining whether he or she finds it interesting, useful, and important.
Control of learning beliefs	Refers to how much the student believes that his or her performance depends on his or her own effort.
Self-efficacy for learning and performance	Represents the student’s beliefs about her/his abilities, as well as his/her performance expectations when faced with a given task.
Test anxiety	Represents a student’s feeling of uneasiness in the context of performance evaluation.
Rehearsal	Refers to the use of strategies of repeating the exposition of information several times with the goal of memorizing the information
Elaboration	Represents the strategies for integrating the learned content with prior knowledge.
Organization	These are the strategies for selecting and integrating the information to be apprehended. Represents the formation of relationships and the construction of meaning of what is being learned.
Critical thinking	It is the use of one’s own logical repertoire to evaluate new information and solve problems.
Metacognitive self-regulation	It is the broad cognition regulation component of metacognition. It refers to the use of strategies for planning, monitoring, and the regulation of cognition itself.
Time and study environment	It concerns the management of time and the organization or selection of a suitable environment for study.
Effort regulation	It is the student’s commitment and ability to control effort and attention during learning situations.
Peer learning	Describes the tendency to interact with peers in order to enhance understanding.
Help seeking	Refers to both the ability to recognize when help is needed and to identify a source that can provide appropriate help.

Psychological services aiming to help college students overcome learning difficulties or enhance self-regulatory processes use the MSLQ as a clinical diagnostic tool and intervention assessment (Casali et al., 2022). MSLQ is also a predictor of academic performance. Credé and Phillips’ (2011) meta-analysis shows that the MSLQ scales that best predict performance in college subjects are effort regulation (ρ = .40), self-efficacy for learning and performance (ρ = .37), and time and study environment (ρ = .31).

Despite its importance, the field of studies shows no evidence that the factor structure of the MSLQ is valid (Liu et al., 2019). Almost all the studies on its factor structure show evidence that refutes the factor structure of the MSLQ and its validity. In addition, almost all studies show methodological inconsistencies that compromise the evidence. The most important methodological inconsistencies are classified into three categories (Table 2). Given that inspection of the structural validity of the MSLQ requires the inclusion of all 81 items in their original form, we disregarded evidence from studies that removed items or tested alternative models derived from exploratory factor analysis (Hands & Limniou, 2023; Villarreal-Fernández & Arroyave-Giraldo, 2022; Wang et al., 2022; Zhou & Wang, 2021).

**Table 2:** Methodological characteristics in studies which dealt with the structural validity of the MSLQ

Study	General factor of SRL		MND and estimator	Models with unacceptable fit
	CFAs realized by the authors	Model with SLR factor		
Adesope et al., 2017	CFA 1: 4 motivation scales CFA 2: 4 strategy scales		MND: NR estimator: NR	X
Alkjarusi et al., 2012	CFA 1: 6 motivation scales CFA 2: 9 strategy scales		MND: NR estimator: ML	X
Bin Dayel et al., 2018	CFA: 6 motivation scales		MND: NR estimator: NR	X
Cho & Summers, 2012	CFA 1: 6 motivation scales CFA 2: 9 strategy scales		MND: NR estimator: NR	X
Chow & Chapman, 2017	CFA 1: 6 motivation scales CFA 2: 9 strategy scales		MND: NR estimator: ML	
Cook et al., 2011	CFA 1: 6 motivation scales		MND: NR estimator: NR	X
Dunn et al., 2012	CFA: 2 strategy scales		MND: NR estimator: NR	X
Hamilton & Akhter, 2009	CFA: 6 motivation scales		MND: reported violated MND estimator: ML	X
Hilpert et al., 2013	CFA: 15 scales jointly	X	MND: NR estimator: NR	X
Jackson, 2018	CFA 1: 6 motivation scales CFA 2: 9 strategy scales CFA 3: 15 scales jointly		MND: NR estimator: ML	X
Karadeniz et al., 2008	CFA 1: 6 motivation scales CFA 2: 9 strategy scales		MND: NR estimator: NR	X
Liu et al., 2019	CFA: 5 strategy scales		MND: reported violated MND estimator: MLR	X
Pintrich et al., 1991	CFA 1: 6 motivation scales CFA 2: 9 strategy scales		MND: NR estimator: NR	X
Rotgans & Schmidt, 2010	CFA 1: 4 motivation scales CFA 2: 9 strategy scales		MND: NR estimator: ML	
Şen et al., 2014	CFA 1: 4 motivation scales CFA 2: 5 strategy scales		MND: NR estimator: NR	

*SLR* self-regulated learning, *CFA* confirmatory factor analysis, *MND* multivariate normal distribution, *NR* not reported, *ML* maximum likelihood, *MLR* maximum likelihood robust

The first inconsistency involves the analysis of the general factor, which represents the self-regulated learning construct itself. This factor is rarely investigated by studies examining the structural validity of the MSLQ. This is an unjustified situation, since the measure of self-regulated learning itself demands the assessment of this construct. Hilpert et al. (2013) and Jackson (2018) are the only studies on the factor structure of the MSLQ that included components of motivation and strategy in the same model, making it possible to inspect the general factor of self-regulated learning. However, only Hilpert et al. (2013) inserted the general factor in the tested models (Table 2). Unfortunately, their study has important limitations regarding the evidence on the general factor. They did not use the 81 questionnaire items as observable variables, but the summed scores of the 15 scales. In summary, they tested two models with the general factor that did not have acceptable fit rates. In one of the models, the general factor was the only latent variable that explained the variance of the scores (one-dimensional model, CFI = .57; RMSEA = .20 and SRMR = .13) while in the other model, the general factor was a second-order latent variable explaining four first-order latent variables (hierarchical model, CFI = .73, RMSEA = .16 and SRMR = .13).

The second inconsistency concerns the use of the correct estimator to carry out the factor analysis of items. To choose the estimator correctly is a central methodological decision since a wrong choice substantially reduces the accuracy of the parameters and increases the likelihood of the model being wrongly rejected (Li, 2016). To properly select an estimator, it is mandatory to inspect the multivariate normality of the items (Li, 2016), and we can see in Table 2 that this is not done or reported by most studies. The maximum likelihood estimator is suitable only when the items follow a multivariate normal distribution, but it is often used by studies without testing for the normality of the data (Li, 2016). Hamilton and Akhter (2009) and Liu et al. (2019) are the only ones who performed this analysis. Although both do not present the statistical results, they report that the data did not exhibit multivariate normality. Even though, Hamilton and Akhter (2009) mistakenly selected maximum likelihood for their analyses. Liu et al. (2019), on the other hand, are the only ones who used a suitable estimator for non-normal data, the maximum likelihood robust estimator.

The third inconsistency refers to the authors’ conclusion that these MSLQ studies show favorable evidence of validity, while almost all the studies show inadequate fit rates for the models tested. Only three studies show models with acceptable fit (Table 2). However, two of these studies exhibit relevant methodological problems that compromise their evidence. Rotgans and Schmidt (2010) found an acceptable fit (CFI = .94 and RMSEA = .05) for a model with the following correlated motivation components: intrinsic goal orientation, extrinsic goal orientation, control of learning beliefs, and self-efficacy for learning and performance. On the other hand, they changed the original MSLQ response scale, so their evidence cannot be transposed to the original MSLQ. Whereas Şen et al. (2014) found an acceptable fit for two models: the motivation model of Rotgans and Schmidt (2010) and the correlated strategy components model (organization, elaboration, metacognitive self-regulation, effort regulation, and time and study environment). Both models showed CFI = .99 and RMSEA = .07. However, this study did not perform factor analysis of items because the authors used the summed scores of the scales as observable variables. In turn, Chow and Chapman (2017) found an acceptable fit for the motivation model, including all six components of the MSLQ correlated (CFI = .95, SRMR = .076 and NNFI = .95). While the evidence from this study is not incorrect, the failure to include the strategy components in the same model compromises the evidence because conceptually, self-regulated learning is an articulation between motivations and strategies.

Therefore, our study aims to correct those inconsistencies and provide solid evidence about the factor structure of the MSLQ. Furthermore, we hope to change this path of inadequate methodological practices by suggesting a correct way to conduct confirmatory factor analysis of items for an adequate analysis of the MSLQ. Also, by pointing out the inconsistencies, we do not intend to deride previous studies. We recognize that in the early 1990s, factor analysis of items was not a well-known technique, with very few software to suitably conduct it. However, this context has changed considerably since the 2010s, and the field of study of self-regulated learning needs to use better methodological practices if it is to build better evidence about its constructs.

## Method

### Participants

The participants in this study come from a convenience sample of 670 students from a Brazilian public university in the field of technology. The majority is male (*N* = 436, 65.1%), with a mean age of 22.8 years (*SD* = 5.2), minimum age of 17 years and maximum age of 52 years, with an interquartile range of 4 years. Considering the age classification suggested by Rae Simpson (2018), this sample has 23 (3.4%) adolescents (< 18 years), 548 (81.8%) young adults (18–25 years), and 99 (14.8) adults (> 25 years). This age variation is typical of many Brazilian universities, in which the majority are young adults, but with the presence of both older adolescents and adults who enter university later in life.

### Instrument

The MSLQ is a self-report questionnaire about self-regulated learning in the context of higher education. It comprises 15 scales, six of them belong to the domain of motivations and nine to the domain of learning strategies. The questionnaire has 81 items: 31 concerning motivations and 50 concerning strategies. Table 1 describes the 15 first-order components of the MSLQ measured by the 15 scales. Each item presents a statement that describes a self-regulatory behavior in the university academic context. The respondent must rate each statement by choosing one option from a seven-point scale: the lowest value indicates that that behavior is not representative of their behavior, and the highest value expresses that that behavior is very representative.

Our study uses a version of the MSLQ with items in Brazilian Portuguese. The motivation items were translated by Ruiz (2005) and are used with the author’s consent. As we did not find a translated version of the strategy items, the 50 items in this domain were translated by the authors of this article following the procedures recommended by the International Test Commission (2017).

### Data collection

The data came from two samples collected in 2018 and 2019 at a public university with students from technology departments. Three instruments were applied to the first sample, in the following order: (1) Metacognitive Monitoring Test, approximately 40 minutes (Gomes et al., 2021b); (2) TAb-Videogame, approximately 10 minutes (Gomes et al., 2020a); (3) MSLQ, approximately 15 minutes. Five instruments were applied to the second sample: (1) Metacognitive Monitoring Test, approximately 40 minutes (Gomes et al., 2021b); (2) TAb-Videogame, approximately 10 minutes; (3) Learning Approaches Scale, approximately 5 minutes (Gomes et al., 2011); (4) MSLQ, approximately 15 minutes; and (5) SLAT-Thinking, approximately 40 minutes (Gomes, 2021a). Data collection followed ethical guidelines (CAAE: 73453317.1.0000.0118). This study was fully supported by the university, which included the director of education and several professors. One of the researchers was invited to give a lecture about the study to the university’s students. Internal disclosures were made by the university, via e-mails and social networks, presenting the purpose of the study and a link that allowed them to answer the instruments on the SurveyMonkey platform. Upon opening the link, the participant only answered the instruments if he or she consented to the Informed Consent Form. All participants were volunteers and did not receive financial compensation. Participants did not have a time limit for answering the tests, so they could rest between them. The answers were automatically saved when the students submitted their answers as they advanced to the next page.

### Data analyses

To correct the inconsistencies of the previous studies, we applied some methodological steps. First, we inspected the multivariate normality of the 81 items of the MSLQ and reported the results. Furthermore, we used item confirmatory factor analysis when assessing the factor structure of the MSLQ, including all 81 items encompassing the six motivations components and the nine strategies components. Finally, we tested models with the general factor representing self-regulated learning.

**Fig. 1:**
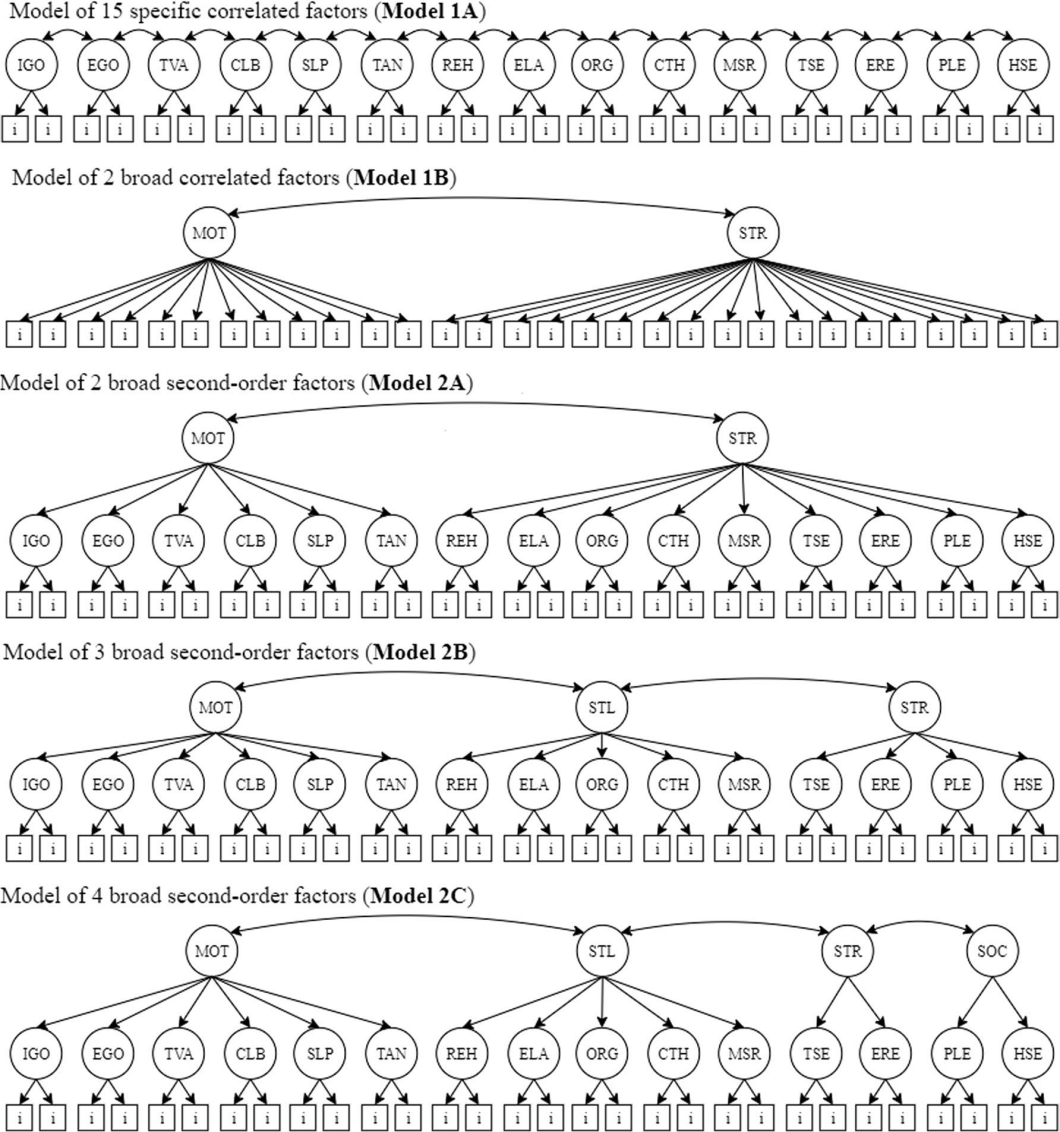
Models tested in steps 1 and 2. Note. IGO = intrinsic goal orientation; EGO = extrinsic goal orientation; TVA = task value; CLB = control of learning beliefs; SLP = self-efficacy for learning and performance; TAN = test anxiety; REH = rehearsal; ELA = elaboration; ORG = organization; CTH = critical thinking; MSR = metacognitive self-regulation; TSE = time and study environment; ERE = effort regulation; PLE = peer learning; HSE = help seeking. MOT = motivation; STR = strategies; LST = learning strategies; RST = regulation strategies; SST = social strategies. All tested models include the 81 items of the MSLQ. The items are represented in the figure by squares. Due to space limitations, not all of them are shown

The data analysis involved planning a few steps. In the first step, we tested two fundamental models stipulated by the theory, including only first-order factors. According to the theory, self-regulated learning can be conceived in terms of 15 specific components or in terms of two broad components, motivation and strategy. So, the first step involves testing two models that represent these two possibilities. The 15-component specific first-order model defines that each of the 15 components in Table 1 respectively explains the items on each scale. This model is represented in Fig. 1 (Model 1A). For space-saving reasons, there are only two rectangles representing the items of each scale; however, it is important to note that each scale has a broad number of items. The first-order two broad factor model, on the other hand, assumes that all 31 motivation items are explained by the broad motivation component while the 50 strategy items are explained by the broad strategy component (Fig. 1, Model 1B).

The second step of the analysis would be performed only if the specific 15 first-order component model had a better fit than the two broad first-order component model and then, the 15 specific components would be explained by different second-order factors. The first model assumes that motivation and strategy, the two correlated second-order factors, explain the first-order factors (Model 2A) and is supported by the arguments of Pintrich et al. (1991). Figure 1 shows how the second-order factors explain the first-order factors. The second model assumes that three correlated second-order factors explain the first-order factors (Model 2B) and is supported by the arguments of Pintrich et al. (1991). The third model assumes that four correlated second-order factors explain the first-order factors (see Fig. 1, Model 2C), which is supported by the meta-analysis of Credé and Phillips (2011).

**Fig. 2:**
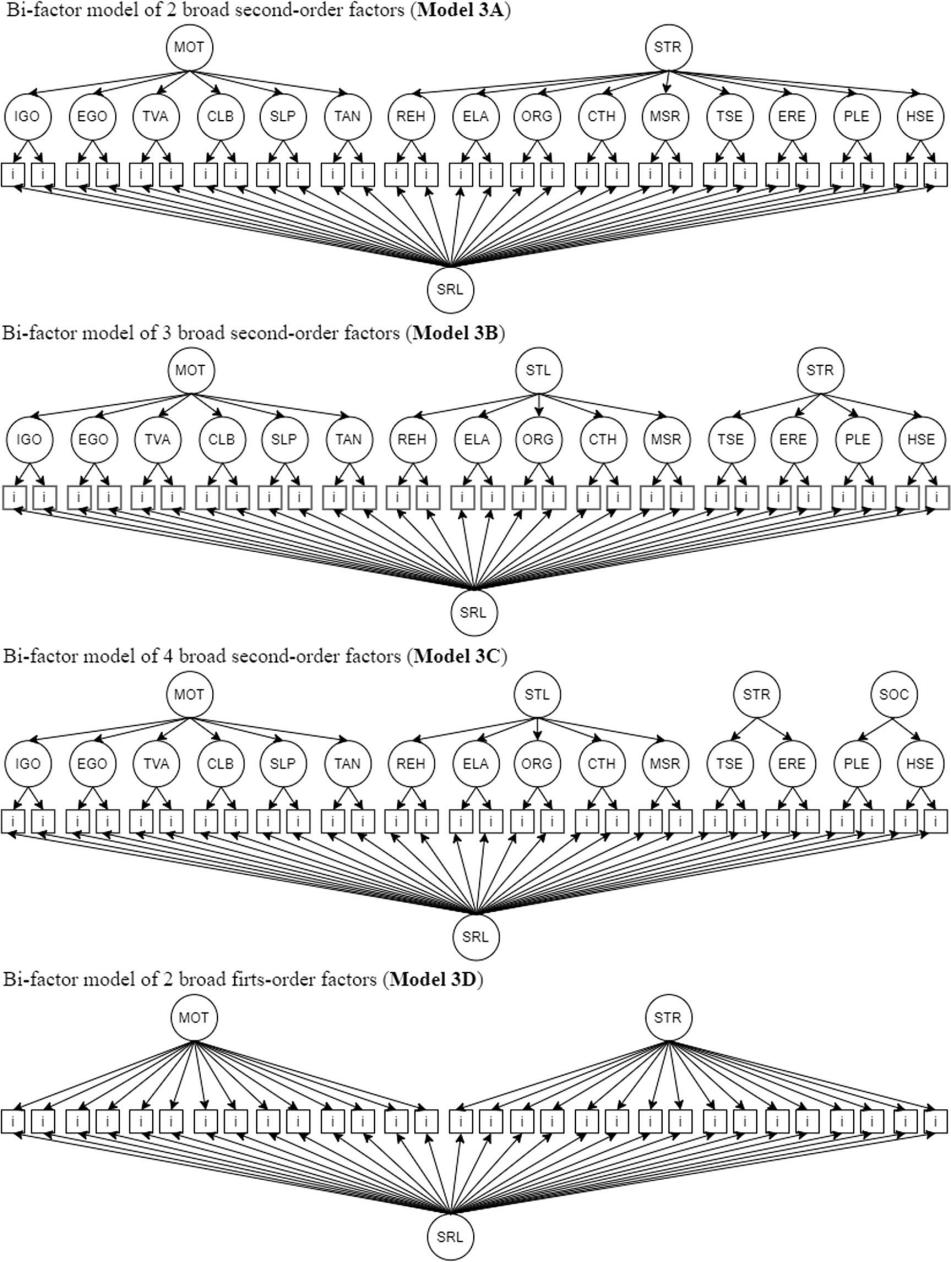
Models tested in step 3. Note. IGO = intrinsic goal orientation; EGO = extrinsic goal orientation; TVA = task value; CLB = control of learning beliefs; SLP = self-efficacy for learning and performance; TAN = test anxiety; REH = rehearsal; ELA = elaboration; ORG = organization; CTH = critical thinking; MSR = metacognitive self-regulation; TSE = time and study environment; ERE = effort regulation; PLE = peer learning; HSE = help seeking. MOT = motivation; STR = strategies; LST = learning strategies; RST = regulation strategies; SST = social strategies. SRL = self-regulated learning. All tested models include the 81 items of the MSLQ. The items are represented in the figure by squares. Due to space limitations, not all of them are shown

The third step includes two possibilities. If the second step had been performed, then the third step would evaluate all the models from step 2, adding a first-order general factor orthogonal to the other components (Fig. 2, Models 3A, 3B and 3C). Otherwise, if the first-order broad two-factor model showed the best fit in the first step analysis, then the general factor would be added to this model and orthogonalized with the other two components (Fig. 2, Model 3D). The third step is crucial, as it assesses the empirical plausibility of the self-regulated learning construct itself.

Since studies on the structural validity of the MSLQ usually use the maximum likelihood (ML) estimator to run confirmatory factor analyses of items, we will also use this estimator to run our models. However, if the result of the Mardia test has a *p*-value well below .05, the weighted least squares means and variance adjusted (WLSMV) estimator, as well as two robust maximum likelihood estimators, the Huber-White maximum likelihood robust (MLR) and Satorra-Bentler’s maximum likelihood mean adjusted (MLM) will be used (Satorra, 2000). Li (2016) argues that the WLSMV estimator is the most appropriate for data where multivariate normality is strongly violated, while robust maximum likelihood estimators would be appropriate for milder violations.

Confirmatory item factor analysis and factor reliability analysis were performed using the packages lavaan, version 0.6.11 (Rosseel et al., 2022), and semTools, version 0.5.5 (Jorgensen et al., 2021) of the R software, version 4.2 (Core Team, 2022). The tested models would be rejected if they had CFI < .90 or RMSEA ≥ .10 (Thakkar, 2020). One model would be considered better than the other when it showed a statistically significant difference in terms of chi-squares and degrees of freedom (Δχ^2^ [Δdf]).

The reliability of the factor scores was assessed using Cronbach’s alpha and McDonald’s omega for the first-order factors, and the general factor as well as Cronbach’s alpha and hierarchical omega (reliabilityL2 semTools R package function) was used for the second-order factors. Factor scores with alpha ≥ .60 and omega ≥ .50 would be considered reliable (Gomes et al., 2020, b, c; Valentini et al., 2015). It should be noted that the cutoff point of .50 for omega was not created for bi-factor models. We know that bi-factor models in which the general factor is orthogonal to the other factors are very demanding models that tend to decrease the factor load of the specific components and consequently decrease their omega. In this case, if the usual cutoff point is .50, it would be plausible to assume that, approximately, an omega of .40 would still be acceptable for bi-factor models.

## Results and discussion

The results shown in Table 3 indicate that the only models tested exhibiting an acceptable fit were those estimated by the WLSMV. These results are not surprising, as the Mardia test suggests a strong violation of multivariate normality of the 81 items of the MSLQ for the sample in this study (skewness = 136,363.4, *p* < .001; kurtosis = 98.7, p < .001). Li (2016) had already argued that robust ML estimators are not appropriate for data that strongly violate multivariate normality, while WLSMV would be the appropriate estimator. The violation of multivariate normality is also not surprising. This result is congruent with the only study that has examined the multivariate normality of the MSLQ (Hamilton & Akhter, 2009; Rotgans & Schmidt, 2010). In the introduction, we showed that only a single study used a robust version of the ML estimator for non-normal data (Liu et al., 2019). Although many studies did not report the estimator (Table 2), it is very likely that maximum likelihood was used since this is the default estimator in many software programs. Considering that model fit tends to worsen with the use of inadequate estimators (Li, 2016), it is very likely that the use of maximum likelihood in the MSLQ structural validity analysis is the main reason for the models’ rejection.

**Table 3:** Fitting the models tested by means of confirmatory factor analysis of items

Step	Model	Estimator	χ^2^[df]	CFI	RMSEA	RMSEA [CI 90%]
1°	15 specific first-order factors (Model 1A)	WLSMV	15,695.377 [3054]	.928	.079	[.077, .080]
		MLR	7666.049 [3054]	.771	.047	[.046, 0.49]
		MLM	7361.588 [3054]	.784	.046	[.045, 0.47]
		ML	8650.788 [3054]	.762	.052	[.051, 0.54]
1°	2 broad first-order factors (Model 1B)	WLSMV	42,489.726 [3158]	.776	.136	[.135, .138]
		MLR	14,400.828 [3158]	.443	.073	[.072, .074]
		MLM	13,934.277 [3158]	.459	.071	[.070, .072]
		ML	16,354.985 [3158]	.438	.079	[.078, .080]
2°	2 broad second-order factors (Model 2A)	WLSMV	23,824.229 [3143]	.882	.099	[.098, .100]
		MLR	8807.994 [3143]	.719	.052	[.051, .053]
		MLM	8457.402 [3143]	.733	.050	[.049, .051]
		ML	9952.699 [3143]	.710	.057	[.056, .058]
2°	3 broad second-order factors (Model 2B)	WLSMV	22,397.998 [3141]	.891	.096	[.095, .097]
		MLR	8691.488 [3141]	.725	.051	[.050, .053]
		MLM	8339.694 [3141]	.739	.050	[.049, .051]
		ML	9811.743 [3141]	.716	.056	[.055, .058]
2°	4 broad second-order factors (Model 2C)	WLSMV	20,755.071 [3140]	.900	.092	[.091, .093]
		MLR	8371.058 [3140]	.741	.050	[.049, .051]
		MLM	8031.979 [3140]	.754	.048	[.047, .049]
		ML	9454.778 [3140]	.731	.055	[.054, .056]
3°	2 broad second-order factors bi-factor (Model 3A)	WLSMV	16,729.393 [3063]	.922	.082	[.080, .083]
		MLR	8079.072 [3063]	.751	.049	[.048, .051]
		MLM	7661.211 [3063]	.769	.047	[.046, .049]
		ML	8990.892 [3063]	.748	.054	[.052, .055]
3°	3 broad second-order factors bi-factor (Model 3B)	WLSMV	No convergence			
		MLR	7809.579 [3063]	.775	.048	[.047, .049]
		MLM	7055.150 [3063]	.799	.044	[.043, .045]
		ML	8313.784 [3063]	.776	.051	[.049, .052]
3°	4 broad second-order factors bi-factor (Model 3C)	WLSMV	15,021.135 [3069]	.932	.076	[.075, .078]
		MLR	7536.807 [3069]	.778	.047	[.045, .048]
		MLM	7184.546 [3069]	.793	.045	[.044, .046]
		ML	8471.851 [3069]	.770	.051	[.050, .053]

*χ*^*2*^ chi-square, *df* degrees of freedom, *CI* confidence interval.

Only some models tested showed an acceptable fit (Table 3). In the first step of model analysis, only the 15-factor specific model (Model 1A) showed acceptable fit (Table 3). This result is relevant from a theoretical standpoint because it supports unprecedented evidence on the structural validity of the MSLQ with the presence of all the components proposed by Pintrich et al. (1991) in the same model. Given the theoretical importance of this model and the fact that its fit was acceptable, we present its factor loadings in Tables 5, 6, 7 and 8, along with the factor loadings of the final model, which will be presented later. The factor loadings of this model can be seen in the third column of Tables 5, 6, 7 and 8. The fourth column of Tables 5, 6, 7 and 8 displays the means, the standard deviations of the factor loadings of each component, as well as their alpha and omega. The results of the 15-factor specific model (Model 1A) suggest that the items of each of the 15 scales of the MSLQ are relevant for gauging the 15 components, as the lowest mean factor loading of the components on the items was .49 and the highest was. 82 (see Tables 5, 6, 7 and 8). The items 33 and 57 of the metacognitive self-regulation factor are exception, as they exhibited a factor loading of less than .30. All other items exhibited a loading equal to or greater than .40. All the MSLQ components are reliable in the 15-factor specific first-order model, showing alpha ≥ .60 and omega ≥ .50. Although alpha is the most widely used reliability index, the psychometric literature has recommended the use of omega or similar since it produces a more accurate estimate of the reliability of latent variables by considering their factor loadings (Cho, 2016). To our knowledge, only one study investigated the reliability of the MSLQ using the omega (Liu et al., 2019). However, the authors found no evidence of structural validity of the instrument. As such, our study is the first to show that components of the MSLQ are reliable via McDonald’s omega. Despite the promising evidence found in the 15 component-specific model, it is relevant to point out that it is not the final model, which would best represent the factor structure of the MSLQ.

In the second step, the four-factor model (Model 2C) was the only one not to be rejected, despite its moderate fit (Table 3). The results of the second step of the model analysis are relevant from a theoretical point of view, as they indicate that the traditional model of two broad components, one of motivation and the other of strategy, does not hold empirically. In short, the results of the second step suggest that the broad component of strategy should be divided into three distinct parts: learning strategy, regulation strategy, and social strategy, according to the theoretical proposition of Credé and Phillips (2011).

In the third step of the model analysis, there was a need for four constraints in the four broad factors model (Model 3C). The factor loadings of items 33, 57, and 62 were constrained to zero on the specific factors because the general factor explained all the common variance of these items. Constraining to zero certain loadings on the specific factors is common in bi-factor models since in these models, the general factor and the specific factors compete to explain the common variance of the items. In this context, when the general factor explains all the item’s common variance, the factor loading of the item on the specific factor can become negative. This does not mean that this item has a negative relationship with the specific factor, but only that all the common variance of this item was explained by the general factor. To correct this negative loading, one should constrain it to zero (Reise et al., 2010). In addition to the constraint applied to the three items, the factor loading of the specific component of critical thinking had its factor loading constrained to zero on the broad component of learning strategies.

The models with the general factor and two and four broad factors (Models 3A and 3C) showed acceptable fit, while the model with the general factor and three broad factors (Model 3B) did not converge. Model 3C, among all models with acceptable fit, was the one with the highest CFI and lowest RMSEA. When we compare this model with the other models with acceptable fit, Models 1A, 2C, and 3A, we can see that Model 3C is the model with the best fit and so, it is considered the final model (Table 4).

**Table 4:** Comparison of Model 3C with other models of acceptable fit

Model	Δχ^2^[df]	*p*
4 broad second-order factors bi-factor (Model 3C)	-	-
15 specific first-order factors (Model 1A)	70.49 [15]	3.647e-9
4 broad second-order factors (Model 2C)	1235.37 [71]	2.2e-16
2 broad second-order factors bi-factor (Model 3A)	522.68 [6]	1.092e-109

Δχ^2^ [df] = chi-square and degrees of freedom difference.

The factor loadings of the first-order components of the final model can be seen in the fifth column of Tables 5, 6, 7 and 8, while the factor loadings of the general factor of this model can be seen in the seventh column of Tables 5, 6, 7 and 8. The factor loadings of the four broad factors in relation to the 15 specific components appear in Table 9.

**Table 5:** Factor loadings and reliability of the motivation factors

Component	Item	Factor loadings and reliability				
		15-factor specific model (Model 1A)		Specific factor of the final model		General factor of the final model
Intrinsic goal orientation	1	.62*	*M* = .60 *SD* = .07 α = .63 Ω = .65	.47*	*M* = .42 *SD* = .20 α = .63 Ω = .31	.43*
	16	.64*		.68*		.41*
	22	.64*		.20*		.49*
	24	.49*		.36*		.33*
Extrinsic goal orientation	7	.78*	*M* = .73 *SD* = .04 α = .76 Ω = .84	.81*	*M* = .67 *SD* = .15 α = .76 Ω = .69	.20*
	11	.72*		.79*		.16*
	13	.68*		.52*		.30*
	30	.75*		.55*		.31*
Task value	4	.66*	*M* = .72 *SD* = .06 α = .82 Ω = .85	.42*	*M* = .51 *SD* = .13 α = .82 Ω = .43	.47*
	10	.66*		.34*		.49*
	17	.69*		.62*		.44*
	23	.72*		.58*		.48*
	26	.75*		.68*		.48*
	27	.82*		.44*		.61*
Control of learning beliefs	2	.84*	*M* = .71 *SD* = .20 α = .74 Ω = .73	.56*	*M* = .71 *SD* = .10 α = .74 Ω = .81	.42*
	9	.53*		.75*		.00
	18	.92*		.79*		.36*
	25	.56*		.75*		.03
Self-efficacy for learning and performance	5	.70*	*M* = .75 *SD* = .10 α = .88 Ω = .94	.50*	*M* = .58 *SD* = .22 α = .88 Ω = .58	.43*
	6	.76*		.75*		.34*
	12	.72*		.58*		.42*
	15	.83*		.76*		.42*
	20	.85*		.69*		.49*
	21	.55*		.09*		.50*
	29	.81*		.67*		.47*
	31	.77*		.58*		.47*
Test anxiety	3	.59*	*M* = .73 *SD* = .15 α = .80 Ω = .88	.58*	*M* = .72 *SD* = .16 α = .80 Ω = .86	−.07
	8	.58*		.56*		.10*
	14	.72*		.71*		.07
	19	.90*		.91*		.07
	28	.87*		.86*		.13*

*M* mean, *SD* standard deviation, *α* Cronbach’s alpha, *Ω* McDonald’s omega, * = statistical significant loadings (*p* < .05).

**Table 6:** Factor loadings and factor reliability of learning strategies

Component	Item	Factor loadings and reliability				
		15-factor specific model (Model 1A)		Specific factor of the final model		General factor of the final model
Rehearsal	39	.78*	*M* = .78 *SD* = .03 α = .79 Ω = .89	.70*	*M* = .67 *SD* = .13 α = .79 Ω = .65	.35*
	46	.82*		.81*		.34*
	59	.74*		.51*		.43*
	72	.77*		.65*		.39*
Elaboration	53	.68*	*M* = .72 *SD* = .07 α = .82 Ω = .88	.36*	*M* = .24 *SD* = .32 α = .78 Ω = .17	.58*
	62	.74*		.00		.70*
	64	.76*		.03		.73*
	67	.61*		.82*		.39*
	69	.81*		.19*		.74*
	81	.74*		.04		.70*
Organization	32	.72*	*M* = .78 *SD* = .07 α = .79 Ω = .85	.63*	*M* = .63 *SD* = .10 α = .79 Ω = .55	.38*
	42	.83*		.62*		.50*
	49	.72*		.51*		.46*
	63	.84*		.76*		.46*
Critical thinking	38	.73*	*M* = .76 *SD* = .03 α = .85 Ω = .87	.48*	*M* = .56 *SD* = .05 α = .85 Ω = .47	.51*
	47	.74*		.56*		.50*
	51	.79*		.56*		.54*
	66	.76*		.62*		.49*
	71	.80*		.60*		.54*
Metacognitive self-regulation	36	.52*	*M* = .49 *SD* = .12 α = .74 Ω = .83	.56*	*M* = .28 *SD* = .20 α = .77 Ω = .32	.36*
	41	.50*		.07		.46*
	44	.51*		.29*		.42*
	54	.50*		.38*		.39*
	55	.58*		.62*		.41*
	56	.52*		.23*		.44*
	57	.27*		.00		.27*
	33	.26*		.00		.26*
	61	.48*		.21*		.40*
	76	.60*		.41*		.44*
	78	.68*		.34*		.56*
	79	.53*		.23*		.46*

*M* mean, *SD* standard deviation, *α* Cronbach’s alpha, *Ω* McDonald’s omega, * = statistical significant loadings (*p* < .05)

**Table 7:** Factor loadings and factor reliability of regulation strategies

Component	Item	Factor loadings and reliability				
		15-factor specific model (Model 1A)		Specific factor of the final model		General factor of the final model
Time and study environment	35	.57*	*M* = .57 *SD* = .14 α = .74 Ω = .78	.24*	*M* = .44 *SD* = .15 α = .74 Ω = .48	.41*
	43	.80*		.50*		.54*
	52	.48*		.54*		.27*
	65	.51*		.26*		.36*
	70	.77*		.49*		.52*
	73	.49*		.34*		.33*
	77	.56*		.66*		.29*
	80	.40*		.50*		.20*
Effort regulation	37	.64*	*M* = .67 *SD* = .06 α = .70 Ω = .75	.72*	*M* = .53 *SD* = .14 α = .70 Ω = .49	.34*
	48	.70*		.39*		.46*
	60	.59*		.51*		.34*
	74	.73*		.50*		.46*

*M* mean, *SD* standard deviation, *α* Cronbach’s alpha, *Ω* McDonald’s omega, * = statistical significant loadings (*p* < .05)

**Table 8:** Factor loadings and reliability of the social strategies factors

Component	Item	Factor loadings and reliability				
		15-factor specific model (Model 1A)		Specific factor of the final model		General factor of the final model
Peer learning	34	.76*	*M* = .81 *SD* = .06 α = .81 Ω = .84	.56*	*M* = .73 *SD* = .16 α = .81 Ω = .67	.39*
	45	.82*		.87*		.26*
	50	.87*		.76*		.39*
Help seeking	40	.55*	*M* = .71 *SD* = .12 α = .71 Ω = .84	.68*	*M* = .59 *SD* = .30 α = .71 Ω = .59	.16*
	58	.68*		.16*		.49*
	68	.84*		.87*		.34*
	75	.78*		.64*		.40*

*M* mean, *SD* standard deviation, *α* Cronbach’s alpha, *Ω* McDonald’s omega, * = statistical significant loadings (*p* < .05)

**Table 9:** Loadings of the 15 specific components on the four broad components of the final model

Broad component	Specific component	Factor loading	Reliability
Motivation	Intrinsic goal orientation	.42*	α = .86 Ωh = .46
	Extrinsic goal orientation	−.12*	
	Task value	.36*	
	Control of learning beliefs	.52*	
	Self-efficacy for learning and performance	.80*	
	Test anxiety	−.40*	
Learning strategies	Rehearsal	.72*	α = .81 Ωh = .86
	Elaboration	.76*	
	Organization	.91*	
	Critical thinking	.00	
	Metacognitive self-regulation	.46*	
Regulation strategies	Time and study environment	.78*	α = .91 Ωh = .76
	Effort regulation	.78*	
Social strategies	Peer learning	.91*	α = .84 Ωh = .90
	Help seeking	.91*	

*α* Cronbach’s alpha, *Ωh* hierarchical omega, * = statistically significant loadings (*p* < .05)

The factor structure of the final model provides new evidence of the empirical identification of the construct of self-regulated learning through the insertion of the general factor in this model. The general factor exhibited a mean factor loading of .39 (SD = .15), which is a good average for bi-factor models, considering that in these models the general factor and the specific factors compete to explain the common variance of the items. However, this does not mean that the general factor had a relevant role in explaining the variance of all MSLQ items. The literature usually points out that an item is relevantly loaded by a factor when it has at least a factor loading of .40 (Howard & Henderson, 2023). Nevertheless, we understand that this cut-off point is not relevant for bi-factor models, given that we have already explained the factor competition to explain the common variance of the items. We did not find in the literature any suggested cutoff point and, therefore, we proposed a cutoff point of .20 to consider that the item had a relevant factor loading. We identified that nine items had a loading of less than .20 on the overall factor (Tables 5, 6, 7 and 8), which is equivalent to about only 10% of the MSLQ items. It should be noted that these items include all five items in the test anxiety component. This result is interesting because it brings a new challenge to the field. From a theoretical perspective, it has been expected by theory that test anxiety items would load negatively on the overall factor, as theory states that the test anxiety component is counterproductive to self-regulated learning (Chow & Chapman, 2017). Regarding the other four items that did not show a factor loading greater than or equal to .20 in the overall factor, it is relevant to perform a future inspection of the content of their sentences to verify whether this content may have contributed to this low loading.

In addition, the overall factor proved to be reliable, as its Cronbach’s alpha was .93 and its McDonald’s omega was .81. From a theoretical point of view, the presence of a valid and reliable overall factor empirically supports the theoretical argument of Pintrich et al. (1991) about the existence of the self-regulated learning construct. In practical terms, the valid and reliable overall factor indicates that the user can apply the MSLQ to perform the measurement of the self-regulated learning construct.

It is interesting to note that the factor loadings of the specific components on the items of each scale remained relevant, despite the presence of a general orthogonalized factor in relation to all other components. This is noteworthy because it indicates that the specific components are important self-regulatory processes that explain some of the variance of the MSLQ items. This is true for 13 of the 15 specific components, of which only items 21 and 58 have factor loadings less than .20. The exceptions are the components of elaboration and metacognitive self-regulation because many of their items are loaded in a relevant way only by the general factor, with loadings lower than .20 on the specific factors. These two components showed inadequate reliability, taking McDonald’s omega as a reference (Table 6). Although the intrinsic goal orientation component has no items with loadings lower than .20, it has few items, also causing inadequate reliability via McDonald’s omega (Table 5). A technical procedure to solve the unacceptable reliability of these three components is to increase the number of items in the scales aimed at measuring them. It is relevant to add items with similar characteristics to those items that load on these components in a relevant way. Until this is done, it is relevant to be cautious about using the scores of these components for clinical practice and psychoeducational diagnoses. We emphasize that future analysis is needed on the sentence contents of items that have low loadings on specific factors, to understand why these items do not adequately represent their components in the presence of the overall factor.

The fact that the final model presents four broad components and not two broad components as strongly claimed by the theory (Pintrich et al., 1991), in part refutes the theory. What would be the broad component strategy is actually three distinct strategies, namely learning strategies, regulation strategies and social strategies. In other words, in the final model there is no broad component strategy and this is what refutes part of the theory.

The factor loadings of the broad components on their specific components support part the theory (see Table 9). The theory states that the specific components of help seeking and peer learning are positive self-regulated learning strategies. The results corroborate this theoretical claim when they show that the broad component of social strategies loads both specific components positively and with strong loadings. The same occurs with the specific components of time and study environment and effort regulation in relation to the broad component of regulation strategies. Regarding the broad component of motivation, it loads positively on the specific components that the theory states are positive self-regulatory motivations. Similarly, it loads negatively on the two self-regulatory motivations that the theory claims are negative.

The factor loadings of the broad component learning strategies on the specific factors partly refute the theory. The specific component rehearsal is loaded in a positive and relevant way by the broad component learning strategies, contrary to the theory’s claim that rehearsal is a strategy negatively associated with the other strategies. This negative relationship between rehearsal and the other MSLQ strategies are declared by self-regulated learning theory because rehearsal is defined by the theory as a low cognitive processing strategy, while the others are defined as high-level cognitive processing (Chow & Chapman, 2017). A possible reason why we found a positive and relevant factor loading between rehearsal and learning strategies in our study may be that students do not perceive and evaluate rehearsal items as mechanical and low processing strategies. The rehearsal items describe memorization and repetition behaviors, but do not necessarily mandate that repetition be exclusively mechanical and unreflective. This is evidenced in the following items from this component: (1) “I memorize keywords to remind me of important concepts in this class” and (2) “When I study for this class, I practice by saying the material to myself over and over”. Although memorization by repetition is often conceived as rote learning in education, it is very possible that repetition is involved with active processes of relationship formation and meaning construction. For example, even by just repeating information, the student may, while repeating the information, articulate the repeated information, integrate it in some way with what he already knows. In short, the student can read the rehearsal MSLQ items and assess that even in “learning by heart” there is high-order cognitive processing. Students might be correct in their self-report. Active subject theories may underestimate information repetition behaviors, making a perhaps naive assumption that if there is repetition, then there is no production of relationships and construction of meaning. There is much evidence from studies on memory in tasks of mere memorization by repetition of stimuli that an individual’s memorization, even by repetition, is involved in information association strategies and that better strategies generate better memorization performance (Carroll & Harris, 2020).

Still on the broad and specific components, we found a peculiar result that the broad component learning strategies have zero loading in relation to the specific component critical thinking. Moreover, the items of the critical thinking scale are well loaded by the general factor. This result suggests that critical thinking is only articulated to the general factor, that is, to self-regulated learning. Of course, we need to understand better why the specific component of critical thinking is not within the scope of direct relations with some broad component but relates only directly to the general factor of self-regulated learning. We do not have a hypothesis about this. Further studies are needed to understand whether this result is a specific feature of our sample or it is a generalizable result.

Our results need to be replicated in other samples for the new evidence to become robust. Despite this warning, the new evidence could have some clinical implications. Throughout the presentation of the results and discussion, we have highlighted several of these implications, but two remain to be discussed. The first of these involves the underutilization of the MSLQ measure. The meta-analyses by Credé and Phillips (2011) and Broadbent and Poon (2015) show that researchers have only used the scores of the 15 specific component measures of the MSLQ. Our evidence shows that both researchers and clinicians can use MSLQ scores for the measure of four broad components of self-regulated learning in addition to the general factor, and need not be restricted to the specific components. The second implication refers to the insufficient reliability of the specific components of metacognitive self-regulation, elaboration, and intrinsic goal orientation. This compromises the evidence for the prediction of these components relative to academic performance. Moreover, it hinders the clinical use of these components, making their diagnosis imprecise. This low reliability is even more serious in the metacognitive self-regulation component since it is one of the three basic components that the field defines as constitutive of self-regulated learning.

## Conclusion

This study brings relevant contributions to the field of self-regulated learning, as it shows new evidence of structural validity of the MSLQ with all its scales in a single model (Pintrich et al., 1991). This study also provides advances for self-regulated learning theory in that it empirically identifies not only the 15 specific components of the MSLQ, but also four broad components and the general factor of self-regulated learning. The results indicate that both the general factor of self-regulated learning and the 12 components of the MSLQ are reliable. It also highlights the relevance of correct estimator selection for confirmatory factor analyses of items. The results showed that the maximum likelihood and its robust estimators are not appropriate for the MSLQ data used in this study. Following Li′s (2016) argument, confirmatory factor analyses of items are most likely to be well estimated by WLSMV when the data strongly violate multivariate normality. It was previously common to assume that if a scale had 7 or 8 points, it would tend to generate a normal distribution, consequently producing a multivariate normal distribution for the items in an instrument. However, this belief did not prove to be adequate. Having 7 points on a scale, as is the case with the MSLQ, is no guarantee that item responses will show multivariate normality. On the contrary, the results found in the literature on the MSLQ indicate multivariate non-normality whenever inspected by researchers. Therefore, it is possible that most studies have been using an incorrect estimator.

In addition to those contributions, the present study also highlights theoretical aspects of self-regulated learning that should be investigated in the future. For example, there is a need for a better understanding of the relationships between the general factor of self-regulated learning and the broad and specific components. So far, we have no information about this, considering the absence of models that include the general factor in the analysis. Besides, we will need to investigate the predictive role of the general factor of self-regulated learning about educationally relevant outcomes such as performance and dropout, as well as the convergent and divergent validity of the general factor regarding certain constructs. We will also need to understand whether the component-specific items of critical thinking and elaboration are really explained by the general factor alone, or whether our result is restricted to the characteristics of our sample. In any case, this result will have to be better understood. The results on the specific component of rehearsal indicate that memorization through repetition strategies is not perceived as superficial by students. This is a result that refutes an important statement of the theory and therefore needs further research.

Although the evidence from this study is promising, it is just the beginning. To the best of our knowledge, our results are the first to bring forward models with acceptable fit to the factor structure of the MSLQ. Furthermore, our results come from a convenience sample consisting of students from the same institution, most of whom are male and young adults. Results based on convenience samples weaken the external validity of the results, but in current practice they often turn out to be the only possible way to do. This limitation in the external validity of our study also occurs in all the studies that examined the factor structure of the MSLQ, which we cite in Table 2. To improve external validity, more studies with diversified samples need to be conducted in order to generate more robust evidence regarding the structural validity of the MSLQ and the reliability of its components. It is relevant that other researchers employ our data analysis strategies, particularly the analysis of multivariate normality, the use of the WLSMV estimator whenever multivariate normality is lacking, and the testing of bi-factor models in which the general factor is present. If our results are reproduced, then the evidence from our study will prove more robust.

This study makes a practical contribution to users of the MSLQ by identifying inadequate reliability for three specific component measures. We do not encourage the use of these scales for clinical practice and psychoeducational diagnosis until the reliability issue is resolved. We recognize that our sample is limited because it is a convenience sample. However, the MSLQ is a widely used test in many parts of the world, serving as a tool for clinical practice and educational diagnosis. Given its importance, it is our point of view that the results of our study are sufficient for researchers to rethink about the use of elaboration, metacognitive self-regulation, and intrinsic goal orientation component measures.

In conclusion, in this paper, we show in detail the mistakes or the limitations present in previous works that invalidated an adequate analysis of the factor structure of the MSLQ. We used a data analysis strategy capable of correcting the mistakes made. We found different models with acceptable fits and defined the best one, calling it the final model. We hope this work can be a guide to the researcher, in order to avoid past mistakes on the structural validity of the MSLQ of being constantly repeated. This study presented a final model that deserves to be investigated in different contexts and samples.

For researchers wishing to test the models applied in our study, we caution that the MSLQ models are complex in terms of the number of parameters, so we suggest using a sample size of at least approximately 1000 students. In addition to the complexity of the models, the WLSMV estimator itself demands a large sample size when run on scales with as many points as in the MSLQ, which has seven points in its scale. Those interested in replicating the tested models can contact the first author of this article and request the scripts run in R software in order to reproduce them.

## Data Availability

The data that support the findings of this study are available from the corresponding author upon request.

## Abbreviations

MSLQ: Motivated Strategies for Learning Questionnaire
ML: maximum likelihood
MLR: maximum likelihood robust
MLM: maximum likelihood estimator with robust standard errors
WLMSV: weighted least squares mean and variance adjusted
CFI: confirmatory fit index
RMSEA: root-mean-square error of aproximation
SRMR: standardized root mean square residuals
MND: multivariate normal distribution
NR: not reported
SLR: self-regulated learning
IGO: intrinsic goal orientation
EGO: extrinsic goal orientation
TVA: task value
CLB: control of learning beliefs
SLP: self-efficacy for learning and performance
TAN: test anxiety
REH: rehearsal
ELA: elaboration
ORG: organization
CTH: critical thinking
MSR: metacognitive self-regulation
TSE: time and study environment
ERE: effort regulation
PLE: peer learning
HSE: help seeking
MOT: motivation
STR: strategies
LST: learning strategies
RST: regulation strategies
SST: social strategies
